# Using methylation data to improve transcription factor binding prediction

**DOI:** 10.1080/15592294.2024.2309826

**Published:** 2024-02-01

**Authors:** Daniel Morgan, Dawn L. DeMeo, Kimberly Glass

**Affiliations:** a Channing Division of Network Medicine, Brigham and Women’s Hospital and Harvard Medical School, Boston, MA, USA; b Department of Biostatistics, Harvard Chan School of Public Health, Boston, MA, USA

**Keywords:** Transcription factor binding prediction, DNA methylation

## Abstract

Modelling the regulatory mechanisms that determine cell fate, response to external perturbation, and disease state depends on measuring many factors, a task made more difficult by the plasticity of the epigenome. Scanning the genome for the sequence patterns defined by Position Weight Matrices (PWM) can be used to estimate transcription factor (TF) binding locations. However, this approach does not incorporate information regarding the epigenetic context necessary for TF binding. CpG methylation is an epigenetic mark influenced by environmental factors that is commonly assayed in human cohort studies. We developed a framework to score inferred TF binding locations using methylation data. We intersected motif locations identified using PWMs with methylation information captured in both whole-genome bisulfite sequencing and Illumina EPIC array data for six cell lines, scored motif locations based on these data, and compared with experimental data characterizing TF binding (ChIP-seq). We found that for most TFs, binding prediction improves using methylation-based scoring compared to standard PWM-scores. We also illustrate that our approach can be generalized to infer TF binding when methylation information is only proximally available, *i.e*. measured for nearby CpGs that do not directly overlap with a motif location. Overall, our approach provides a framework for inferring context-specific TF binding using methylation data. Importantly, the availability of DNA methylation data in existing patient populations provides an opportunity to use our approach to understand the impact of methylation on gene regulatory processes in the context of human disease.

## Introduction

The binding of transcription factor (TF) protein complexes to gene regulatory regions is one of the primary mechanisms influencing gene expression. DNA recognition sequences, commonly summarized in position weight matrices (PWMs), can be compared with genomic DNA to identify potential TF binding sites, providing a first-order approximation of how TFs regulate genes. Although PWM-based analyses can be useful, they are known to be very limited in scope [1,2]. For example, PWM-independent methods, such as a gapped-kmer model, can be used to predict TF binding in a way that better captures additional genomic signals, such as the impact of flanking co-factors [1,2]. However, all these approaches work by identifying patterns in genomic DNA and must therefore be integrated with additional data to provide context-specific TF binding predictions.

Many successful approaches have been developed to predict context-specific TF binding using epigenetic data, primarily by combining PWM-based motif predictions with chromatin accessibility data [3–5], including histone modifications [6,7], ATAC-sequencing [8], and DNase hypersensitivity [9–11]. However, chromatin accessibility data can be costly to generate and is challenging to collect at scale in part due to sample handling requirements. As a result, most large cohort studies with patient samples do not collect chromatin data. Thus, approaches for predicting context-specific TF binding using chromatin accessibility data are not well suited for performing population-level analyses of TF binding in the context of environmental factors or disease.

Methylation is an epigenetic modification that reflects the dynamic regulatory changes the genome undergoes in response to the environment. Methylation can be collected on banked blood samples, lending itself to large-scale population study of epidemiological epigenetics. Due to these technical advantages as well as its known association with environmental factors and complex diseases, DNA methylation is often assayed in human disease cohorts. There are two main methods for generating site-specific methylation data: whole-genome bisulfite sequencing (WGBS) and array data. WGBS provides genome-wide information regarding methylation. In contrast, methylation arrays, such as the Illumina 450k and EPIC (850k) arrays, are high throughput assays that capture only a subset of methylation sites. However, these sites are selected, in part, based on their likelihood of involvement in gene regulation; therefore, these data are well-suited for predicting context-specific TF binding when chromatin information is unavailable.

Methylation of gene regulatory regions (enhancers and promoters) is generally believed to inhibit TF binding, although the direction of effect may vary by tissue [12]. In particular, the presence (or absence) of methylation alters the DNA structure, impacting the ability of a TF to bind to the DNA [13]. Once bound, a TF can remove methylation from its bound sites [14]. Therefore, in general, absence of methylation is correlated with open chromatin and TF binding. However, this is a highly nuanced relationship. For example, Lui et al. [15] identified a subclass of TFs that appeared to have an affinity for binding to methylated CpG sites. Genome-wide studies have also suggested that there are three classes of TF binding in response to CpG methylation (affinity, restriction, and neutral). For example, Yin et al. [16] used SELEX profiling to systematically characterize TF binding preferences in the context of CpG methylation and classified TFs based on whether methylation had no effect, increases, decreases, or otherwise impacts TF binding affinity [16]. Relatedly, Wang et al. performed manual literature curation to develop a database containing information about methylated DNA binding activities [17]. Importantly for this work, although several studies have used methylation data to predict TF binding, these have focused only on specific cell line-TF combinations [18–22]. To our knowledge, no study has ever used methylation data to predict genome-wide TF binding across a broad range of TF and cell line combinations.

Here, we investigate using methylation data to predict context-specific TF binding. We assess our approach using WGBS, methylation array, and TF ChIP-seq data from six cell lines. We also use annotation information to distinguish the impact of methylation on TF binding in the context of various genomic elements. Finally, we present an option for generating predictions for TF binding when motif locations do not overlap with the methylation sites captured by a specific technology. As the scientific community continues to generate large amounts of methylation data, especially methylation array source data, we believe our approach will support the development of reliable and accurate models of gene regulatory mechanisms, including regulatory network inference. Because array methylation data are available in many large-scale human population studies [23], our approach could be applied to capture evidence for TF binding relevant to human disease.

## Materials and methods

**Figure 1.**
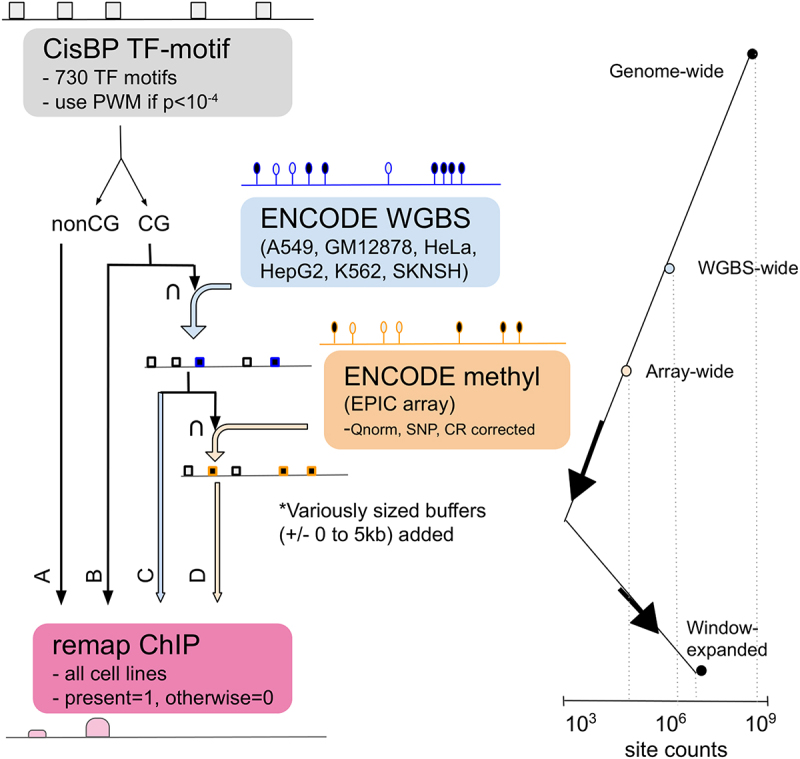
Intersection schema between data. schematic workflow of how we intersected the data in our analysis. The intersection of all three data types with ChIP-seq data (CG motif ∩ WGBS ∩ array ~ ChIP) is the primary analysis presented in this manuscript and shown in Figures 2–3. This narrow view is then expanded by increasing the genomic region associated with each predicted transcription factor binding site (Figure 4).

We used FIMO (Finding Individual Motif Occurrences) [24] to scan the hg38 genome for human TFs based on PWMs selected from CIS-BP [25] and curated by the MEME suite (downloaded 21 February 2018). This analysis identified a list of genomic regions containing potential binding sites for each TF (based on FIMO’s default cut-off of *p* < 1×10^−4^); each genomic region also has an associated PWM-score. For an individual TF, the length of these genomic regions is constant; however, this length varies from 7 to 20 bp depending on the TF. We analyse subsets of these genomic locations (also referred to as motif locations) by intersecting with other available genomic data using bedtools [26]. In this analysis, we considered two genomic regions as overlapping if they shared at least one base pair in common (default setting in bedtools). Our approach is illustrated in Figure 1. For the final list of TF PWMs used in our analyses, see Table S1.

**Figure 2.**
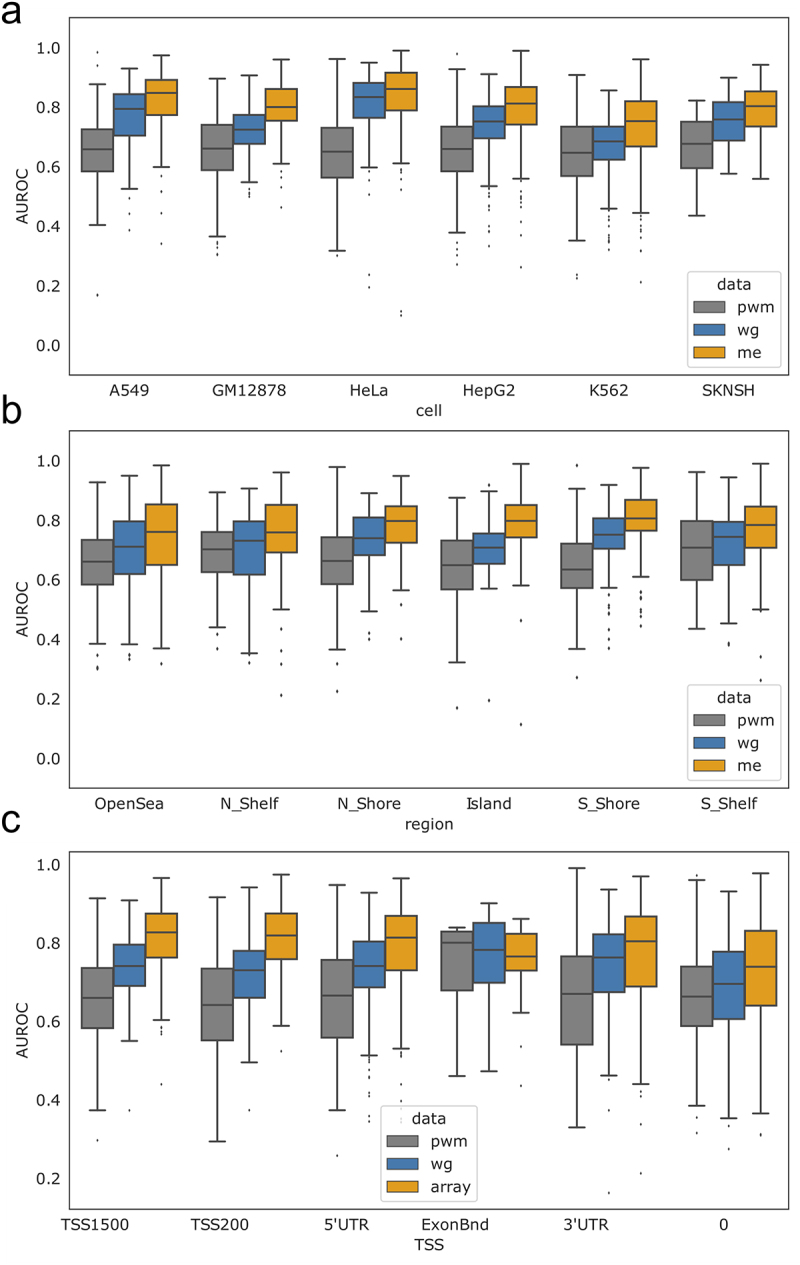
AUROC comparisons across cell lines and genomic annotations. (a) Boxplots representing the distribution of TF AUROC scores for each cell line. All AUROCs improve using methylation-based scoring compared to PWM-based scoring at a significance level of *p* < 2×10^−3^. AUROC scores for individual TFs are shown in figure S5. (b) Boxplots representing the distribution of TF AUROC values when assessed within different genomic regions, indicating that the overall improvement in predicting TF binding can be attributed to region-specific improvement. figure S6 has TF specific breakdowns. (c) Boxplots representing the distribution of TF AUROC values when assessed in the context of gene regulatory regions, indicating that the overall improvement in predicting TF binding can be attributed to improvements in or near the TSS. Note that the ExonBnd has an order of magnitude less associated motif locations. 0 refers to unlabelled. figure S7 has TF specific breakdowns. figure S8 includes additional predictions in the context of CpG islands and promoters. pwm = PWM (position weight matrix); me=methyl array; wg=WGBS.

**Figure 3.**
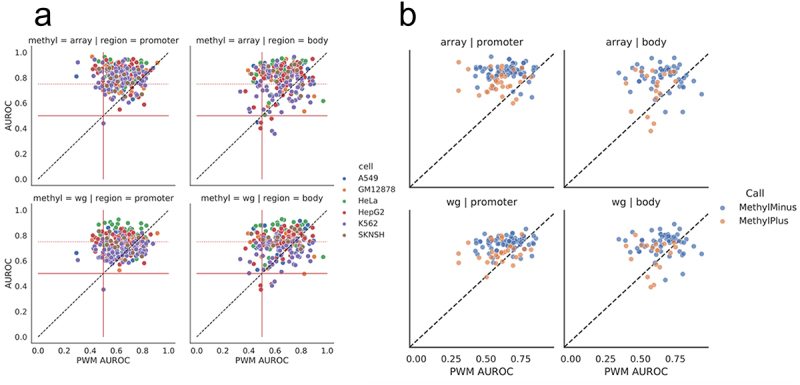
Performance of individual TFs in different genomic regions. (a) Performance of PWM-based scoring for each TF and cell line (each point is a unique TF/cell line combination), compared to scoring based on methylation type (WGBS or methylation array) and genomic context (gene body or promoter). Solid red lines at 0.5 are displayed to highlight TFs with no methylation associated signal. A dotted red line at 0.75 is plotted along the methylation-specific y-axis. Several CEBP family members lie below 0.5 AUROC and are listed in supplemental figure S7F. (b) Performance of PWM-based scoring for each TF (each point is a unique TF), compared to the performance based on methylation type (WGBS or methylation array) and genomic context (gene body or promoter). The colour of the circles indicates whether the TF belongs to the MethylMinus (blue) or MethylPlus (yellow) class as defined in yin et al. In contrast to (a), in these plots each point is a unique TF averaged across cell lines to match cell line agnostic labels from yin et. al.

Next, we downloaded previously processed ChIP-seq data for six cell lines (A549, GM12878, HeLa, HepG2, K562, SKNSH) from ReMap [27] (for more information, see Supplemental Materials and Methods). Similar to motif locations, the length of the genomic regions (ChIP-seq peaks) in these data varies, with a median length of 259 bp and an interquartile range of [179 bp, 394 bp]. For each PWM, we compared its identified motif locations to cell line-specific ChIP-seq peak locations for the PWM’s assigned TF. We note that the number of TF PWMs (and associated ChIP experiments) we were able to evaluate for each cell line varied, from 17 (35) in SKNSH to 105 (357) in K562 (Table 1). For a given TF and cell line, this process resulted in two values associated with each motif location: a score from FIMO based on the PWM match and a binary (0/1) score based on whether a ChIP-seq peak overlapped with that location in a specific cell line. Genome-wide, we observed that approximately one-third (32.8%) of ChIP-seq peaks overlapped with at least one corresponding TF motif location; however, only 1.8% of motif locations overlapped with at least one corresponding ChIP-seq peak (Figure S1). This illustrates the importance of leveraging additional data to determine which motif locations correspond to actual context-specific TF binding.

**Table 1.** Counts of intersecting regions across omics. While generally on the same order of magnitude, all cell lines have a slightly different number of assessed regions (motif locations) across the various omics. Intersecting the regions available for each of the omics determines the number of transcription factors and motif locations assessed in our analyses. Rows in which the first column (row title) is bolded indicate the number of elements (TF locations) being assessed; other rows show the median AUROC across all the TFs evaluated in a cell line. The letters in the first column of this table references Figure 1.

	A-549	GM12878	HeLa-S3	Hep-G2	K-562	SK-N-SH
**A. & B. Number of Motif Locations (total)**	661,411,492 regions					
No. ChIPed TFs/No. ChIP experiments	35/73	62/162	39/99	76/263	105/357	17/35
Motif Locations ~ ChIP PWM AUROC (median value)	0.736564	0.69786	0.701851	0.655201	0.673095	0.719813
**C. Motif Locations ∩ WGBS (total)**	58,607,924 regions					
**Motif Locations ∩ WGBS | ChIPed TF**	5,730,179	10,430,926	7,911,257	24,991,439	30,755,708	3,259,752
Motif Locations ∩ WGBS ~ ChIP PWM AUROC (median value)	0.6757	0.646664	0.700702	0.651493	0.631897	0.6142
Motif Locations ∩ WGBS ~ ChIP WGBS AUROC (median value)	0.814172	0.748682	0.829486	0.719322	0.654999	0.6459
**C. Motif Locations ∩ WGBS > 10**	31,627,874	39,351,126	40,895,066	35,252,422	38,613,480	28,277,399
Motif Locations ∩ WGBS > 10 ~ ChIP PWM AUROC (median)	0.675005	0.639158	0.707343	0.667426	0.629679	0.6026
Motif Locations ∩ WGBS > 10 ~ ChIP WGBS >10 AUROC (median)	0.837216	0.76158	0.849123	0.744336	0.636023	0.6113
**Main Analysis**						
**Illumina array**	794,388 regions (850K)					
**D. Motif Locations ∩ WGBS > 10 ∩ array | ChIPed TF**	225,579	378,540	287,154	373,840	635,836	110,656
Motif Locations ∩ WGBS > 10 ∩ array ~ ChIP WGBS AUROC (median)	0.8578	0.737264	0.890802	0.759793	0.653297	0.7445
Motif Locations ∩ WGBS > 10 ∩ array ~ ChIP WGBS >10 AUROC (median)	0.857005	0.720758	0.893343	0.757426	0.654679	0.7466
Motif Locations ∩ WGBS > 10 ∩ array ~ ChIP array AUROC (median)	0.857005	0.817258	0.914843	0.813226	0.706379	0.7358

We used the binary ChIP-seq score as a gold standard to calculate TF and cell line specific AUROC (Area Under the Receiver-Operator Characteristic curve) scores. Since methylation generally occurs at CpG dinucleotides, we parsed TF motif locations into two mutually exclusive groups, the first consisting of locations not containing CpG sites and the second consisting of locations containing CpG sites. We then calculated the AUROC separately using each set of motif locations. Although for some TFs only a small fraction of motif locations overlapped with CpG sites, we observe no significant differences in the AUROCs associated with these two sets, indicating that the motif locations which we can score using methylation data (which, by definition, will contain a CpG) is not a biased subset (Figure S2).

Next, we downloaded WGBS and EPIC array methylation data for six cell lines (A549, GM12878, HeLa, HepG2, K562, SKNSH) from ENCODE [28] (accessed 15 January 2020; for more information, including data processing information and accessions for the downloaded data see Supplemental Materials and Methods). We determined the correlation between these two methylation technologies, focusing specifically on CpGs within TF motif locations, and observed technical artefacts for low read depths (Figure S3A-B). Based on this analysis, for each cell line we filtered to include methylation sites with a WGBS read depth ≥10. This cut-off allowed us to maximize correlation between the technologies while minimizing loss of sites. We then used these data to score TF motif locations that contain at least one methylation site measured in both the WGBS data (among sites with a read depth ≥ 10) and in the array methylation data. Since increased methylation has been observed to be associated with decreased TF binding [12,20], for each motif location we calculated a ‘Methyl-score’ (*m*) that is inversely related to the methylation measure.

(1) $$m=1-\frac{1}{n}\overset{n}{\underset{i}{\sum }}\beta_{i}$$

In Equation 1 β_**i**_ represents the percent methylation value for any CpG site *i* that overlaps with the motif location. In this way, we upweight motif locations that overlap with hypomethylated CpGs (*m* approaches 1) and down-weight motif locations that overlap with hypermethylated CpGs (*m* approaches 0). Based on Equation 1, only CpGs within a binding site can directly impact the Methyl-score; however, when multiple CpGs with measured methylation values are present within the same motif location (*n > 1*), we take their average. We also explored alternative ways of scoring motif locations that overlap with multiple CpGs, including taking the median, minimum, or maximum (Figure S3C). This process results in cell line-specific Methyl-scores for a subset of motif locations – those that overlap with CpGs with at least 10 reads in WGBS that are also included in the array data. We note that this greatly limits the number of motif locations being assessed, primarily due to the array data only measuring a subset of all possible methylation sites (Figure 1, Table 1). However, it also enables us to directly compare the results between WGBS and the more widely available EPIC array data. We also note that focusing on motif locations with methylation information slightly improved overlap with ChIP-seq data; across the tested TF cell line combinations, the median percentage of motif locations in this subset that overlapped with a corresponding ChIP-seq peak was 5%.

We use this reduced set of TF motif locations together with EPIC array annotations, which have been shown to be reliable when benchmarked against external annotation tools [29,30], to investigate if genomic context impacts the inhibitory role of methylation on TF binding. In the annotation analysis, we determine the primary annotation of each methylation site, and then score motif locations by averaging across CpGs that have a specific associated primary annotation (*A*):

(2) $$\hat{m}=1-\frac{1}{n}\overset{n}{\underset{i\in A}{\sum }}\beta_{i}.$$

Finally, to put our observations into the context of the current literature, we extracted results from the Yin et. al. study [16], which used SELEX data to characterize the effect of methylation on the binding of human TFs. From this paper, we focused on the 292 TFs categorized by the authors as either MethylPlus (prefers methylated CpG; *N = 175*) or MethylMinus (prefers unmethylated CpG; *N = 117*) and disregarded TFs labelled as having ‘multiple effects,’ ‘Little Effect,’ or ‘no CpG.’ Of these 292 TFs, 66 were also evaluated in our motif scan and had ChIP-seq data in at least one cell line (Table S1).

## Results

### Predicting TF binding using methylation data

We leveraged four data types in our analysis: (1) genome-wide motif locations derived by running FIMO on PWMs from CIS-BP and curated by MEME, (2) WGBS and (3) EPIC array methylation for six cell lines from ENCODE, and (4) ChIP-seq peaks for these same cell lines from ReMap. We observed no statistically significant differences in predicting TF binding between motif locations containing CpGs and those devoid of CpGs when evaluating performance using the PWM-score (Figure S2). Furthermore, we find that the methylation levels of CpGs assayed using array and WGBS technologies are highly correlated, although we observe a lack of resolution for low-coverage CpG sites in the WGBS (Figure S3A-B). Thus, we thresholded to only include CpGs from WGBS data for which the sequence read depth was greater than or equal to 10. We note that this does not significantly reduce the number of methylation sites considered in our main analysis since most sites (>75%) that are measured by the Illumina array have a read depth greater than or equal to 10 in the WGBS data.

Next, for each of the six cell lines, we scored motif locations that overlapped with at least one methylation site that is assayed by both the WGBS and array technologies. We compared methods of aggregating methylation information when multiple assayed CpGs overlap with the same motif location. We observed that using different aggregative approaches (mean, median, min, max) to score motif locations that have more than one associated CpG gives highly correlated results (Figure S3C). Based on this analysis, when multiple methylation sites are associated with the same motif location, that location is given a single value based on averaging the methylation levels of all associated sites (Equation 1); for clarity we refer to this as the Methyl-score. This process resulted in two cell line-specific Methyl-scores for each characterized motif location: one based on methylation values from WGBS and one based on methylation values from the Illumina EPIC array.

For each TF motif and cell line, we compared these Methyl-scores with ChIP-seq data (Figure S4) and quantified how accurately they predict TF binding. The distribution of the results, as quantified using the AUROC, is shown in Figure 2(a). TF-specific AUROCs and other comparisons can be found in Figure S5. We observe a low level of prediction when scoring motifs locations based on their original PWM-score (AUROC = 0.55–0.65) consistent with previous findings [31]. However, when scoring motif locations based on methylation data, we observe much higher accuracy, with an overall AUROC increase of 0.2 (Figure 2a) and individual TF increases of 0.1–0.4 (Figure S5C). These results strongly suggest that methylation data can be used to predict context-specific TF binding when chromatin accessibility data are not available. Interestingly, we also observe that prediction levels are often similar for related TFs. For example, CEBP TFs tend to have marginal AUROC scores (~0.55) while many members of the ETS TF family have AUROC scores around 0.8 (Figure S5D). While both WGBS and array methylation information improve our ability to predict TF binding compared to PWM-scores, the extent of improvement is significantly greater for the array data compared to the WGBS (p-value 1 × 10^−36^ based on a paired t-test). This may be due to the fact that methylation values obtained using sequencing technology are dependent on the coverage of individual sites and tend to peak at exactly zero or one (Figure S4). This impacts performance since it is impossible to statistically distinguish locations that are given identical Methyl-scores.

Next, we used Illumina methylation array annotations for CpG Islands, shores, and shelves [32] and evaluated if the methylation information associated with different genomic contexts play distinct roles in predicting TF binding. To do this, we calculated context-specific Methyl-scores for each motif location (Equation 2). For each TF and cell line, we selected the subset of motif locations associated with a particular annotation and benchmarked these scores by comparing with ChIP-seq; this resulted in three AUROC values (for the PWM-score, WGBS Methyl-score, and array Methyl-score) for each TF, cell line, and annotation combination. We then averaged AUROC values across TFs assessed in multiple cell lines, resulting in one value per TF for each annotation. The distribution of the results for annotations related to gene body proximity and TSS region are shown in Figure 2(b,c). TF specific results and other comparisons can be found in Figure S6-S7.

This analysis indicates that methylation information associated with regulatory regions increases predictive performance, with the highest AUROC improvement occurring when assessing motif locations within CpG islands, followed by the shores and the shelves [32]. Only marginal improvement is observed for motif locations annotated to open sea regions (Figure 2b). Similarly, methylation sites annotated to regions near the transcriptional start site (TSS) appear to greatly enhance our ability to predict TF binding, with the highest AUROC increase observed when using motif locations annotated to promoter regions (Figure 2c). In contrast, methylation sites annotated to exons had a predictive capacity no better than the original PWM-score. Combining these annotations, we find that motif locations within CpG islands that overlapped with promoters had a similar predictive capacity as motif locations within CpG islands that were not in promoters; however, the predictive capacity of motif locations that were in promoter regions but not CpG islands was slightly lower (Figure S8).

### Context-specific patterns in TF binding predictions

Given the plasticity of the epigenome, we next investigated if predictive performance varies for individual TFs across different cell lines and genomic contexts. To do this, for each TF and within each cell line, we scored and determined the predictive capacity of motif locations in promoters versus the gene body (as defined by Illumina) [32] and compared to the predictive capacity of the PWM-score (Figure 3a). We find that for most, but not all, individual TFs and cell lines, TF binding is better predicted when scoring motif locations using methylation information (Figure 3a above diagonal), although for each scoring scheme and context there are a small number of poor predictions (AUROC values around 0.5). We also note that the AUROC values are significantly higher when evaluating motif locations associated with promoter regions (TSS200 and TSS1500) as compared to the gene body (3’ UTR, 5’ UTR and Exon Boundary) when using the array data (two-sided t-test p-value = 0.002). However, this is not the case for WGBS data (two-sided t-test p-value = 0.177). This further supports our previous observation that promoter-specific methylation, especially that captured using array technology, is enhancing our ability to correctly predict TF binding (see Figure 2c).

We also observe that, primarily in the context of the gene body, the binding of some TFs is better predicted using the original PWM-score rather than the Methyl-score (Figure 3a below diagonal). In particular, there are several TFs, mostly belonging to the CEBP family (Figure S7F), for which the presence of DNA methylation appears to enhance, rather than inhibit TF binding in a specific context (AUROC values of about 0.4). These findings are consistent with our previous identification of CEBP TFs as having an overall poorer performance using the Methyl-score (Figure S5D). We note that binding of these TFs is also generally poorly predicted by the PWM-score – all but one has an AUROC based on the PWM-score of less than 0.6, with half having a PWM-based AUROC of approximately 0.5 or less. Therefore, it is difficult to draw a specific conclusion for these TFs. However, these results are consistent with previous observations that some CEBP proteins can bind to both unmethylated and methylated versions of their primary DNA recognition sequence, and that methylation may enable them to bind to additional, non-canonical binding sites [33–35].

Although decreased methylation (which corresponds to an increased Methyl-score; see Equation 1) is generally correlated with open chromatin and TF binding, methylation at a specific site may have other, more nuanced effects on TF binding. Therefore, we next compared our results to the ‘MethylMinus’ (prefers unmethylated CpG) and ‘MethylPlus’ (prefers methylated CpG) TF classes identified in Yin et al. [16], which used SELEX data to determine TF affinity to bind to methylated DNA. We hypothesized that TFs in the MethylMinus group might have better performance in our analysis since TFs in this group prefer to bind to unmethylated CpGs, consistent with the definition of the Methyl-score (Equation 1). To perform this analysis, similar to the approach we took in Figure 2(b,c), we first averaged AUROC values for TFs assessed in multiple cell lines, resulting in one value for each TF. Then, for the subset of TFs with a designated class in Yin et al. (~40%), we plot the AUROCs based on the Methyl-score versus the PWM-score (Figure 3b). We then compared the AUROC values between TFs designated as MethylPlus and MethylMinus. We find that both the array and WGBS derived Methyl-scores exhibit a differential performance between these classes within promoters (p-values equal to 4.89 × 10^−7^ and 1.14 × 10^−7^, respectively, based on paired t-test) and, to a lesser extent, the gene body (p-values 5.79 × 10^−4^ and 1.24 × 10^−4^), with TFs in the MethylMinus group having overall higher AUROC values. Finally, we specifically determined the Yin et al. classification for TFs that our previous analyses highlighted as potentially differentially impacted by DNA methylation (Figure S5D). We found high concordance; the CEBPs in our analysis were classified by Yin et al. as MethylPlus, while ETS TF family members were classified as MethylMinus (Table S1).

### Leveraging methylation information from proximal CpGs

Most TF motif locations do not directly overlap with a CpG site covered in the array data and therefore were excluded from our primary analysis. For example, of more than 661 million genomic locations identified in our original motif scan, only 31.6 million overlapped with a CpG methylation site covered in the A549 WGBS with a read depth of more than 10; this shrunk to only 225.6 thousand locations when we restricted to methylation sites contained in the array data and motif locations that corresponded to potential TF binding sites for which we had ChIP-seq data (Table 1). Given this limited coverage, we next investigated our ability to predict TF binding when using methylation values from CpGs that are nearby, but do not necessarily directly overlap with, a given motif location. Although we acknowledge that only methylation within a bound site can directly impact TF binding, we hypothesized considering these additional methylation sites would allow us to maximize information since proximal CpGs are known to be highly correlated [36]. Therefore, we added a window before and after each identified motif location, essentially enlarging the genomic region covered by each. Next, we intersected methylation sites from WGBS and array data with motif locations that had been expanded based on various window sizes, and scored these locations based on Equation 1. We investigated adding windows of ± 5, 10, 20, 50, 100, 250, 500, 1000, 2500, or 5000 bp. These window sizes allow us to investigate the scale at which CpG methylation is predictive of TF binding. To ensure comparable results, we scored the same set of motif locations for each window.

**Figure 4.**
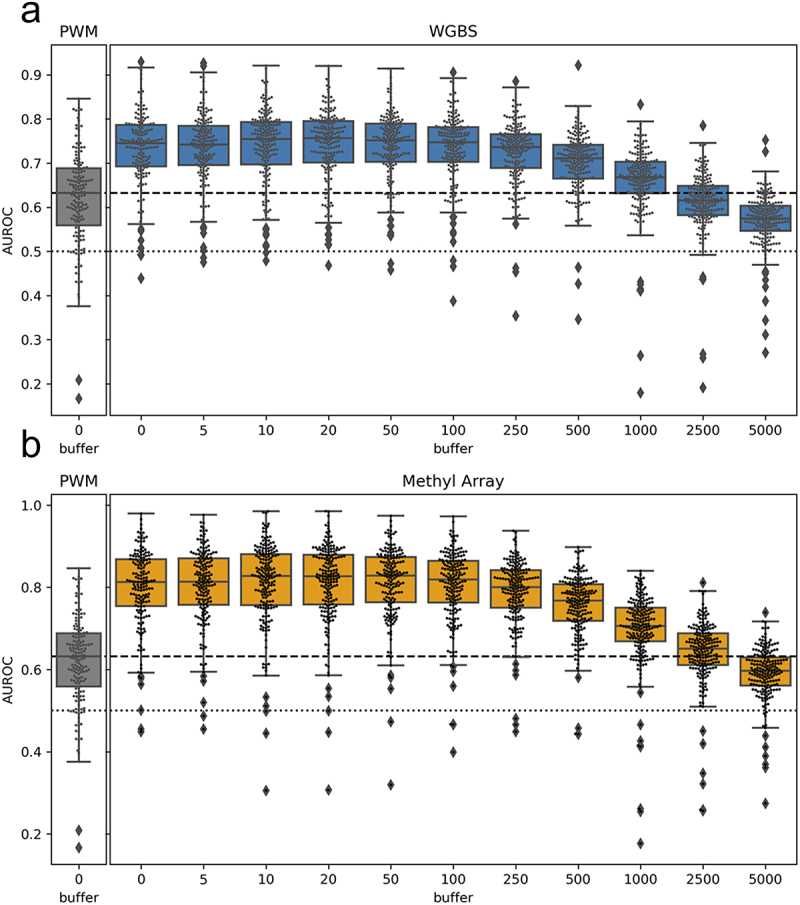
Performance when using nearby CpGs to score motif locations: boxplots representing the distribution of all TF AUROC scores in all cell lines when the methyl-score is derived from nearby CpGs. The x-axis indicates the window (or buffer) around the motif locations that was used. A buffer of 0 indicates direct overlap of CpG sites with the motif locations, as was done for the analyses shown in Figure 3–4. We observe similar performance up to a window size of about ± 100bp, after which there is a drop-off in predictive performance. figure S9 shows that the average difference in methylation levels for pairs of CpGs within a given range has a complementary pattern as what we observe here.

Figure 4 shows the distribution of AUROC values across all TFs and cell lines when using the PWM-score, the baseline Methyl-score when using no window (equivalent to the results from Figure 2a), and the various Methyl-scores obtained when expanding motif locations based on each of the window sizes. We find that the distribution of AUROC values is highly stable up to a window size of ± 100bp. This may be due to local correlation in methylation levels across short genomic distances, as has been previously described [20] and which we also observe in our own data (Figure S9). Overall, this analysis indicates that we can use methylation information to predict TF binding for motif locations that do not directly overlap with a CpG site assayed by a specific technology. Instead, methylation sites that are within ± 100 bp of a motif location can be used to score that location and predict TF binding.

## Discussion

In this manuscript, we evaluate using methylation data to predict genome-wide *in vitro* TF binding. To do this, we scored predicted TF binding sites using methylation levels for CpGs captured both by WGBS and on Illumina EPIC microarrays across six cell lines; we then benchmarked by comparing with ChIP-seq data for TFs from these same cell lines. We found that our methylation-based score consistently performed better at predicting TF binding compared to the original PWM-score and was most predictive of *in vitro* TF binding when the CpGs used to score binding sites were annotated to gene regulatory regions, including CpG islands, shelves, and shores, as well as promoters (as defined by Illumina [32]). Finally, we incorporated methylation levels from CpGs that are nearby, but not necessarily overlapping with, a predicted binding site and observed consistent performance when using information for CpGs that are up to ± 100bp away from predicted TF binding sites. Together with our observation that the highest predictive performance is for TF binding sites located in gene regulatory regions, we believe our analysis sets the stage for using high-throughput methylation arrays to estimate gene regulatory networks.

The main strength of our analysis is that it provides a high-level, comprehensive assessment of how methylation data can be used to provide condition-specific prediction of TF binding. Many previous studies have aimed to predict genome-wide TF binding using chromatin accessibility data [3–11]. We recognize that it is unlikely that our methylation-based method has significant independent predictive power compared to these existing approaches. In addition, since DNA methylation levels are highly correlated with open chromatin, it is not surprising that we found that DNA methylation in a range around a TF binding site has similar predictive power as individual CpG dinucleotides. However, in contrast to methods that use open chromatin information, our approach can be applied to the extensive amount of existing methylation data generated in large cohort disease studies [23], which almost never collect chromatin data.

Our results are also consistent with the current understanding of how methylation impacts TF binding. For example, we observed that the Methyl-score had minimal predictive capacity for a small subset of TFs, many of which were CEBPs. CEBPβ has previously been found to bind nearly equally well to both methylated and unmethylated versions of its binding site [34], and both CEBPα and CEBPβ have been shown to bind to a methylated version of the consensus CREB binding site [33], indicating that methylation levels may have only minimal impact on CEBP binding to the DNA. For methylation sensitive TFs such as CEPBs, we believe that alternative PWMs, such as the ones in databases such as MethMotif [21,37], may improve predictive performance. This is an important future direction for our group. We also investigated whether our results were consistent with previously observed TF binding behaviour in the context of methylation, as reported by Yin et al.[16]. We found only minimal differences in our ability to predict binding between TFs assigned to the classes defined in this paper, although there was a small, but statistically significant, increase in the AUROC values associated with the set of TFs Yin et al. identified as preferring to bind to unmethylated DNA. Differences in platform technology and overall analysis approach contribute to the minimal absolute difference in predictive performance between TFs in the classes identified in this paper.

We recognize that there are several limitations in our approach. First, we focused on methylation data from cell lines. Although this allowed us to systematically benchmark our results using a large amount of ChIP-seq data, methylation data in cell lines likely have distinct differences from that of primary cells and that gathered for complex diseases or from tissue samples. Second, we focused on TFs for which there is a known PWM and for which we had ChIP-seq data; this may be a biased subset. Third, all but one of the cell lines used in this study is of malignant origin. We chose these cell lines since there is a large collection of associated TF ChIP-seq data that we could use to assess our approach; however, going forward, it will be important to repeat these assessments for non-malignant cells [38]. Fourth, our context-specific analysis may be limited by our use of annotations from the Illumina methylation array [32]. However, our results are consistent with the current understanding of the regulatory role of DNA methylation and the wide use of the Illumina platform will allow us to interpret our findings in light of other studies in the field. Furthermore, a large amount of methylation array data has already been generated for multiple longitudinal and disease cohorts using these platforms, primarily using banked blood samples [23]. Although DNA methylation patterns are cell-type specific [38], our analysis gives us confidence that we can use our approach to characterize important aspects of TF regulation and its evolution across disease progression using these data. This is an important future direction for our group [31].

Finally, we note that by focusing on CpGs assayed in both WGBS and methylation array data, we were only able to assign Methyl-scores to a small subset of potential TF binding sites. Although this approach allowed us to directly compare these technologies, it also severely restricted our search space. However, we performed an analysis that indicated that we could score TF motif locations that do not directly overlap with an assayed CpG. We note that considering CpGs up to ± 100bp away from predicted TF binding sites would allow us to score over ten times more TF binding sites compared to only using direct overlap; this includes approximately one-third of the predicted TF binding sites in gene promoter regions, areas that typically have high coverage on methylation microarrays. We also observed that using data collected using microarray technology improves TF binding prediction more than data collected using bisulfite sequencing. This may be due to Poisson error in methylation levels for sites with relatively low sequencing depth. In our analysis, we used a read depth cut-off that minimized loss of sites; a more stringent cut-off would likely improve predictions made with the Methyl-score based on WGBS technology.

In summary, in this manuscript, we investigate scoring predicted TF binding sites using methylation data. We show that methylation-based scores significantly improve our ability to predict experimental (ChIP-seq) TF binding compared to PWM-scores. Importantly, our results are robust across TFs, cell lines, and methylation platforms. We believe using methylation data to improve TF binding prediction provides a framework that can be used to better understand the mechanistic and regulatory drivers of biological systems.

## Supplementary Material

Supplemental_Material_Methods_and_Figures_12112023.pdf

TableS1_TF_AUCvals_Table.xlsx

## Data Availability

All data used in this paper are publicly available. Locations where the data can be downloaded and accession numbers are provided in the Supplemental Material.
