# Supplementary material for: Using methylation data to improve transcription factor binding prediction
Source: Epigenetics. 2024 Feb 1;19(1):2309826. doi: 10.1080/15592294.2024.2309826 (PMC10841018; doi:10.1080/15592294.2024.2309826)
Supplement: Supplemental_Material_Methods_and_Figures_12112023.pdf [file KEPI_A_2309826_SM3193.pdf]

Supplemental Materials and Methods

Analysis Code can be found at: https://github.com/dcolinmorgan/mili_benchmark/

ChIP-seq Data via ReMap:

Preprocessed information about ChIP-seq binding locations for transcription factors (TFs) was downloaded from ReMap[1] (https://remap.univ-amu.fr/download_page). We downloaded the "all peaks" file for *Homo sapiens* based on ReMap2020. From this file we extracted binding sites for TFs for which we had motif information (see below) and that were assayed in cell lines for which we had methylation data (A549, GM12878, HeLa, HepG2, K562, SKNSH; see below). These ChIP-seq locations were intersected with predicted motif locations and used to assess the predictive performance of the PWM-score as well as the array-based and WGBS-based Methyl-scores.

TF Motif Information

We downloaded TF PWM (position weight matrix) information from the meme suite (http://meme-suite.org/meme-software/Databases/motifs/motif_databases.12.15.tgz; accessed: September 14, 2016) and extracted a curated set of Cis-BP human PWMs (file: motif_databases/CIS-BP/Homo_sapiens.meme). We then used FIMO (Finding Individual Motif Occurrences)[3] to scan the hg38 genome for these PWMs, retaining matches with a $p<10^{-4}$ (default cutoff). All TFs and motifs considered are listed in **Table S1**.

WGBS Data via ENCODE:

We identified WGBS (whole genome bisulfite sequencing) data in ENCODE for six cell lines (A549, GM12878, HeLa, HepG2, K562, SKNSH); links to these data can be found using the following URL:

https://www.encodeproject.org/matrix/?type=Experiment&status=released&assay_slims=DNA+methylation&biosample_ontology.classification=cell+line&assay_title=WGBS&biosample_ontology.term_name=A549&biosample_ontology.term_name=K562&biosample_ontology.term_name=GM12878&biosample_ontology.term_name=HeLa-S3&biosample_ontology.term_name=HepG2&biosample_ontology.term_name=SK-N-SH

For each cell line we downloaded preprocessed data by selecting files of type "bed bedMethyl" and output state "methylation state at CpG". Specific accessions for the downloaded data include ENCSR481JIW (A549), ENCSR890UQO (GM12878), ENCSR550RTN (HeLa), ENCSR881XOU (HepG2), ENCSR765JPC (K562), and ENCSR145HNT (SKNSH).

meArray Data via ENCODE:

We identified Illumina EPIC array data in ENCODE for six cell lines (A549, GM12878, HeLa, HepG2, K562, SKNSH); links to these data can be found using the following URL:

https://www.encodeproject.org/matrix/?type=Experiment&status=released&award.project=ENCODE&files.platform.term_name=Illumina+Infinium+Methylation+EPIC+BeadChip&biosample_ontology.term_name=A549&biosample_ontology.term_name=K562&biosample_ontology.term_name=GM12878&biosample_ontology.term_name=HeLa-S3&biosample_ontology.term_name=HepG2&biosample_ontology.term_name=SK-N-SH&assay_title=DNAme+array

For each cell line we downloaded idat data files for both the red and green channels, and used the preprocessNoob() and getBeta() functions in the minfi R package[2] to extract beta values for CpGs. Specific accessions for the downloaded data include ENCSR481JIW (A549), ENCSR890UQO (GM12878), ENCSR550RTN (HeLa), ENCSR881XOU (HepG2), ENCSR765JPC (K562), and ENCSR145HNT (SKNSH).

Supplemental Figure and Table Legends

Figure S1: Overlap between ChIP-seq and motif locations. The percentage of ChIP-seq locations that overlap with one or more motif location (left) and the percentage of motif locations that overlap with one or more ChIP-seq locations (right). For each cell line, the distribution across the assessed TFs is shown.

Figure S2. Comparing PWM-based predictions between motif locations with and without a CpG. (A) A comparison of the PWM-score's ability to predict TF binding (ChIP-seq) when limited to motif locations that contain, or do not contain, a CpG. A T-Test comparing performance within each of the cell lines confirms no significant difference. P-value for A549: 0.233128, GM12878: 0.566850, HeLa: 0.235387, HepG2: 0.336910, K562: 0.127878, and SKNSH: 0.365327. **(B)** Bar chart showing the percentage of motif locations containing a CpG before intersecting with the methylation data.

Figure S3. WGBS sequence depth analysis for a representative example: CEBPG in K562. (**A)** Scatter plots comparing WGBS to array values when restricting to assayed methylation sites that overlap with CEBPG motif locations and that also have a WGBS read depth greater than 0, 10, or 20. **(B)** Histograms of WGBS values for these same methylation sites. **(C)** Density curves (via fit kernel density estimates) comparing various methods for aggregating methylation β values, broken into groups depending on the number of methylation sites that overlap with the predicted TF motif locations: 2, 3, 4, 5, or 6+ CpGs per motif location. We note that the patterns in these plots are similar for other transcription factors and cell lines.

Figure S4: Distribution of the WGBS and array-based Methyl-scores across all assessed motif locations, separated by motif locations that overlap with a corresponding ChIP-seq peak (red line) and those that don't (black line).

Figure S5. Details supporting main analysis. (A) Same plot as shown in **Figure 2A.** Each point is a TF-cell line combination. **(B)** Table showing the T-test statistic and p-value when comparing the AUROC distributions for WGBS and methyl array to AUROC distributions for the PWM-score. **(C)** Heatmap showing individual cell line specific TF performance. Note that the rows are ordered based on the percentage of total motif locations that contain a CpG (see **Figure S2B**), indicating these results do not depend on the overall level of overlap with CpGs in the genome. **(D)** AUROC performance for each TF (averaged across cell-lines) when scoring motif locations based on WGBS and methyl array. We observe similar performance between the technologies, although related transcription factors often have similar levels of performance. Groups of related TFs are highlighted. Underlines indicate TFs classified by Yin et al as MethylPlus (red line) or MethylMinus (blue line). **(E)** Distribution of the difference in AUROC performance for each TF (averaged across cell-lines) comparing WGBS minus PWM-based scoring (blue) and comparing Methyl Array minus PWM-based scoring (orange). **(F)** Distribution of the difference in AUROC comparing WGBS and Methyl Array showing that a Methyl-score derived from the methylation array data generally performs better than one derived from WGBS data.

Figure S6. Detailed analysis based on genomic region. (A) Same plot as shown in **Figure 2B**. **(B)** Statistical analyses comparing the distribution of AUROC scores for each pair of regions when scoring motif locations using

PWM, WGBS, or methylation array. Shades of red indicate significant differences based on a T-test and associated p-value. **(C)** Cell line specific versions of panel (A). **(D)** Heatmap of specific TF performance averaged across cell lines per genomic region annotation. Blue indicates an AUC>0.5 while red indicates AUC<0.5. **(E)** Differential AUROC between methylation array and WGBS indicating that scoring based on methylation array generally performs better than scoring based on the WGBS data.

Figure S7. Detailed analysis based on TSS annotations. (A) Same plot as shown in **Figure 2C**. **(B)** Statistical analyses comparing the distribution of AUROC scores for each pair of TSS annotations when scoring motif locations using PWM, WGBS or methylation array. Shades of red indicate significant differences based on a T-test and associated p-value. **(C)** Cell line specific versions of panel (A). **(D)** Heatmap of specific TF performance averaged across cell lines per TSS annotation. Blue indicates an AUC>0.5 while red indicates AUC<0.5. **(E)** Differential AUROC between methylation array and WGBS indicating that scoring based on methylation array generally performs better than scoring based on WGBS data. **(F)** Transcription factors whose context- and cell line specific binding is better predicted using the PWM compared to methylation data and whose methylation-based predictive performance has an AUROC <0.5. Many of these are CEBP family members.

Figure S8: Distribution of AUROC scores when scoring motif locations using CpGs annotated to (1) both CpG Islands (Islands/Shores/Shelves) and gene promoters (TSS200 or TSS1500), (2) CpG Islands and not gene promoters, and (3) gene promoters but not CpG Islands.

Figure S9: Average difference in the β values for all pairs of CpGs within a given range. For consistency with the main text, only CpGs that were assayed by both methylation array and in WGBS with a read depth of at least 10 were used.

Table S1: The overall AUROC performance of all evaluated TFs based on the PWM-score, the array-based Methyl-score, and the WGBS-based Methyl-score. AUROCs are averaged across cell lines.

Supplemental References

1. Cheneby J, Menetrier Z, Mestdagh M, Rosnet T, Douida A, Rhalloussi W, Bergon A, Lopez F, Ballester B. ReMap 2020: a database of regulatory regions from an integrative analysis of Human and Arabidopsis DNA-binding sequencing experiments. Nucleic Acids Res. 2020;48(D1):D180-D8. Epub 2019/10/31. doi: 10.1093/nar/gkz945. PubMed PMID: 31665499; PMCID: PMC7145625.
2. Aryee MJ, Jaffe AE, Corrada-Bravo H, Ladd-Acosta C, Feinberg AP, Hansen KD, Irizarry RA. Minfi: a flexible and comprehensive Bioconductor package for the analysis of Infinium DNA methylation microarrays. Bioinformatics. 2014;30(10):1363-9. Epub 2014/01/31. doi: 10.1093/bioinformatics/btu049. PubMed PMID: 24478339; PMCID: PMC4016708.
3. Grant CE, Bailey TL, Noble WS. FIMO: scanning for occurrences of a given motif. Bioinformatics. 2011;27(7):1017-8. Epub 2011/02/19. doi: 10.1093/bioinformatics/btr064. PubMed PMID: 21330290; PMCID: PMC3065696.

Supplemental Figures

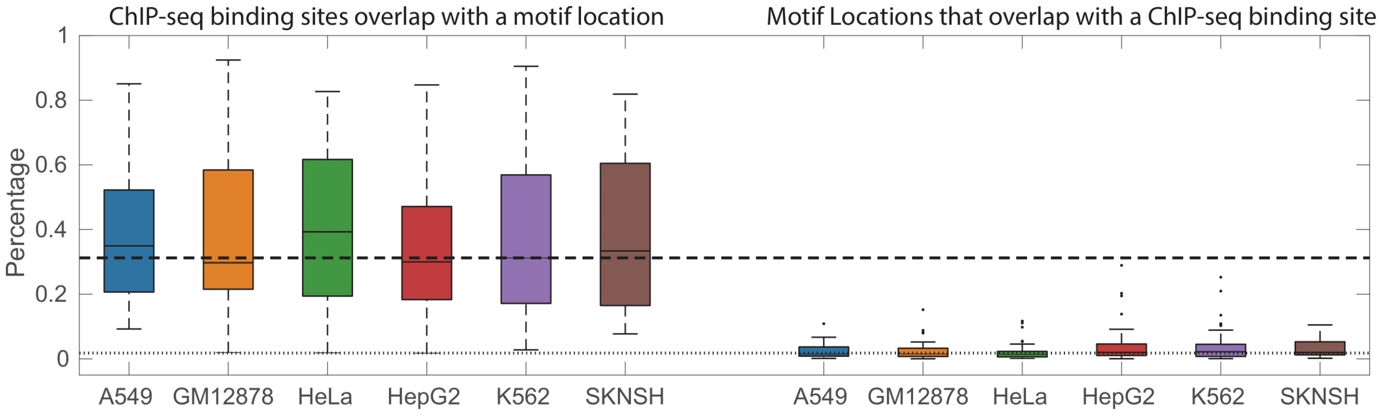

Figure S1: Overlap between ChIP-seq and motif locations. The percentage of ChIP-seq locations that overlap with one or more motif location (left) and the percentage of motif locations that overlap with one or more ChIP-seq locations (right). For each cell line, the distribution across the assessed TFs is shown.

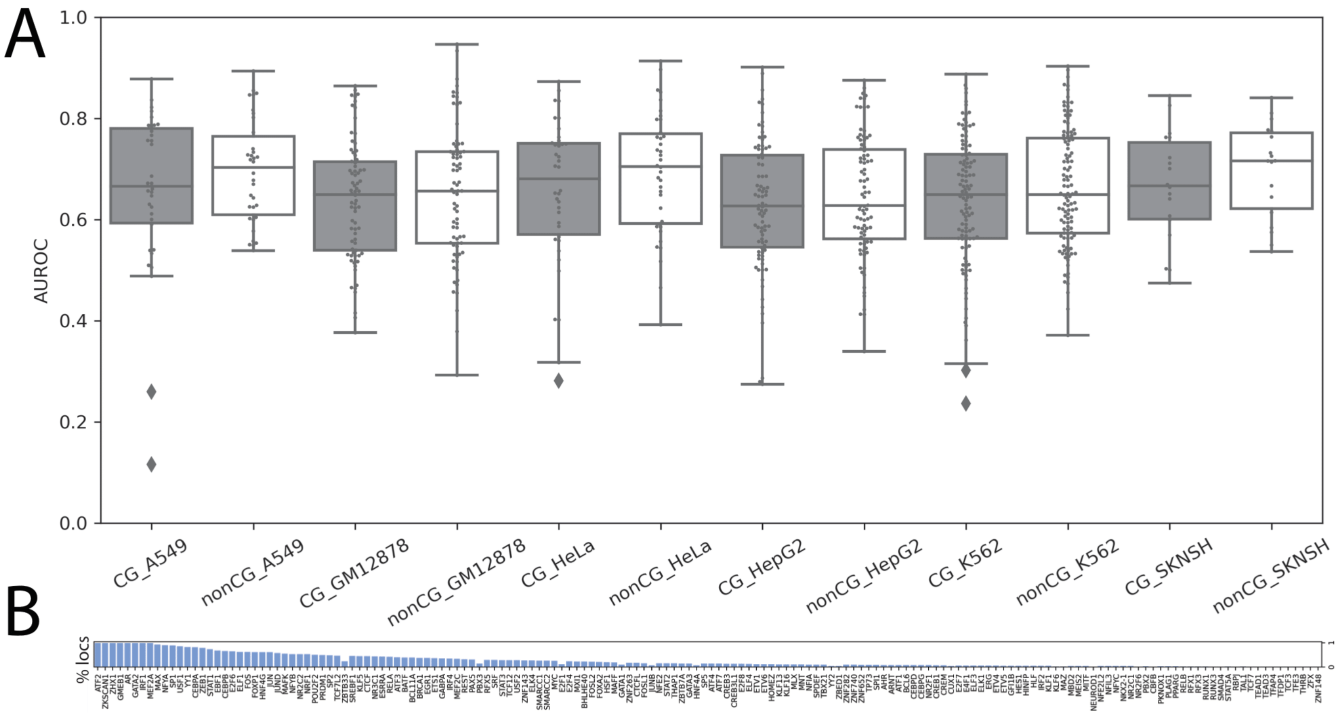

Figure S2. Comparing PWM-based predictions between motif locations with and without a CpG. (A) A comparison of the PWM-score's ability to predict TF binding (ChIP-seq) when limited to motif locations that contain, or do not contain, a CpG. A T-Test comparing performance within each of the cell lines confirms no significant difference. P-value for A549: 0.233128, GM12878: 0.566850, HeLa: 0.235387, HepG2: 0.336910, K562: 0.127878, and SKNSH: 0.365327. **(B)** Bar chart showing the percentage of motif locations containing a CpG before intersecting with the methylation data.

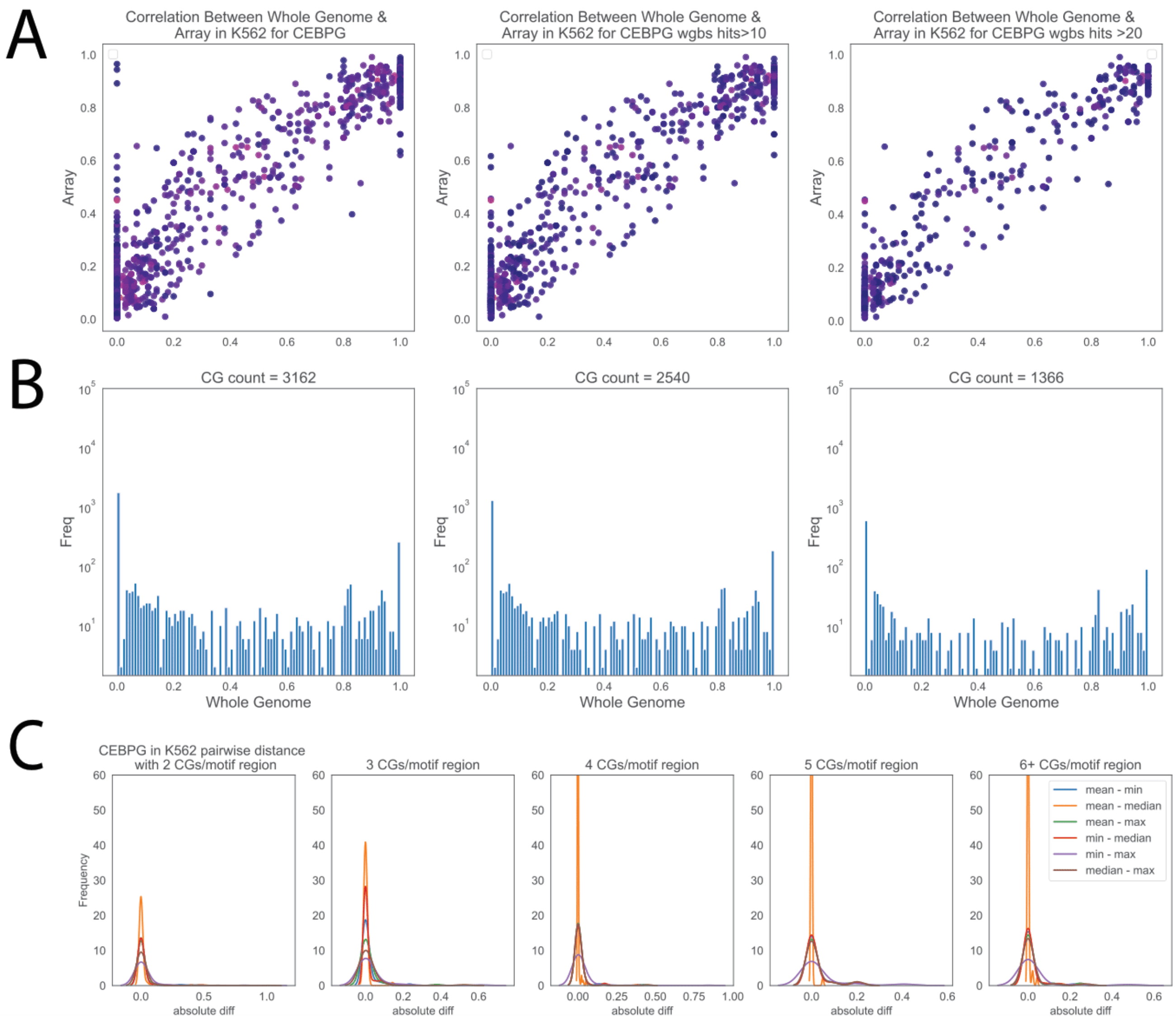

Figure S3. WGBS sequence depth analysis for a representative example: CEBPG in K562. (**A)** Scatter plots comparing WGBS to array values when restricting to assayed methylation sites that overlap with CEBPG motif locations and that also have a WGBS read depth greater than 0, 10, or 20. **(B)** Histograms of WGBS values for these same methylation sites. **(C)** Density curves (via fit kernel density estimates) comparing various methods for aggregating methylation β values, broken into groups depending on the number of methylation sites that overlap with the predicted TF motif locations: 2, 3, 4, 5, or 6+ CpGs per motif location. We note that the patterns in these plots are similar for other transcription factors and cell lines.

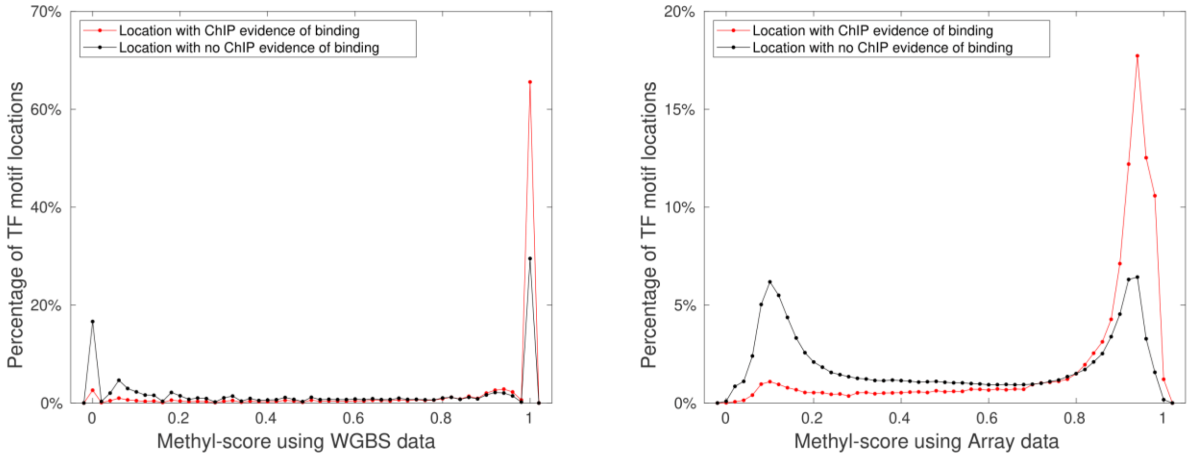

Figure S4: Distribution of the WGBS and array-based Methyl-scores across all assessed motif locations, separated by motif locations that overlap with a corresponding ChIP-seq peak (red line) and those that don't (black line).

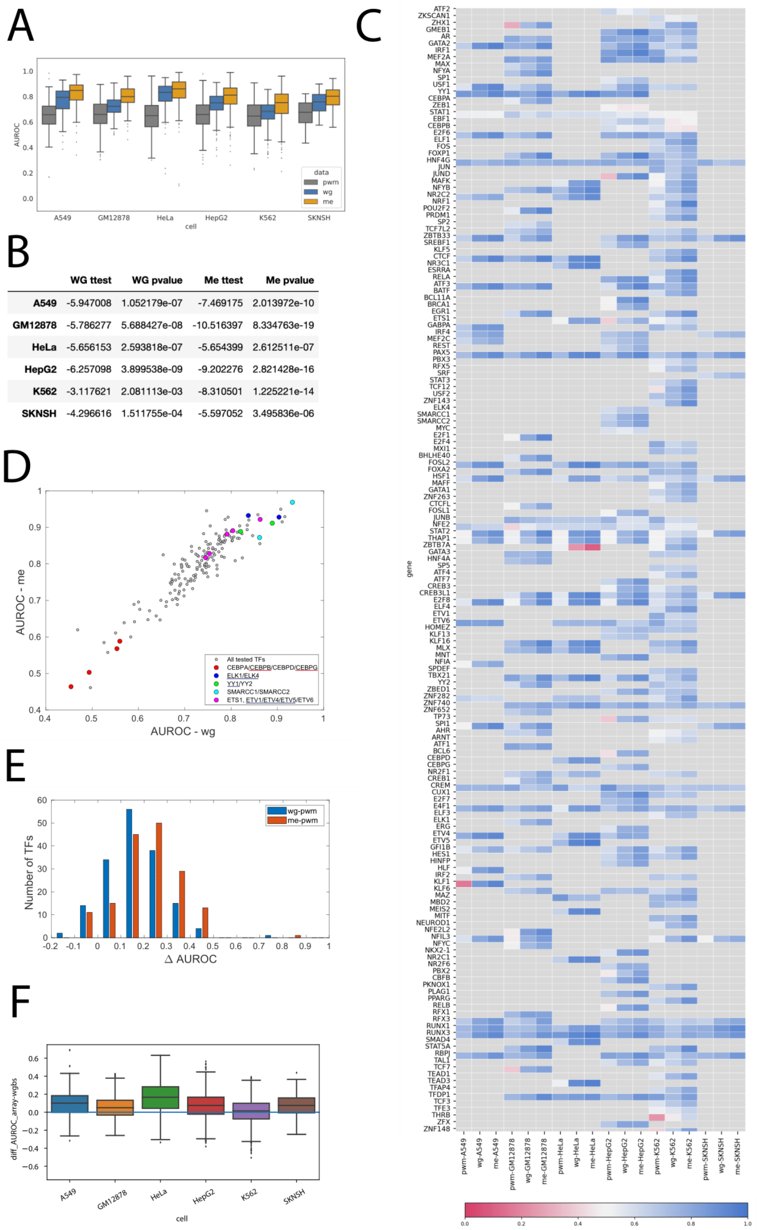

	WG ttest	WG pvalue	Me ttest	Me pvalue
**A549**	-5.947008	1.052179e-07	-7.469175	2.013972e-10
**GM12878**	-5.786277	5.688427e-08	-10.516397	8.334763e-19
**HeLa**	-5.656153	2.593818e-07	-5.654399	2.612511e-07
**HepG2**	-6.257098	3.899538e-09	-9.202276	2.821428e-16
**K562**	-3.117621	2.081113e-03	-8.310501	1.225221e-14
**SKNSH**	-4.296616	1.511755e-04	-5.597052	3.495836e-06

Figure S5. Details supporting main analysis. (A) Same plot as shown in **Figure 2A.** Each point is a TF-cell line combination. **(B)** Table showing the T-test statistic and p-value when comparing the AUROC distributions for WGBS and methyl array to AUROC distributions for the PWM-score. **(C)** Heatmap showing individual cell line specific TF performance. Note that the rows are ordered based on the percentage of total motif locations that contain a CpG (see **Figure S2B**), indicating these results do not depend on the overall level of overlap with CpGs in the genome. **(D)** AUROC performance for each TF (averaged across cell-lines) when scoring motif locations based on WGBS and methyl array. We observe similar performance between the technologies, although related transcription factors often have similar levels of performance. Groups of related TFs are highlighted. Underlines indicate TFs classified by Yin et al as MethylPlus (red line) or MethylMinus (blue line). **(E)** Distribution of the difference in AUROC performance for each TF (averaged across cell-lines) comparing WGBS minus PWM-based scoring (blue) and comparing Methyl Array minus PWM-based scoring (orange). **(F)** Distribution of the difference in AUROC comparing WGBS and Methyl Array showing that a Methyl-score derived from the methylation array data generally performs better than one derived from WGBS data.

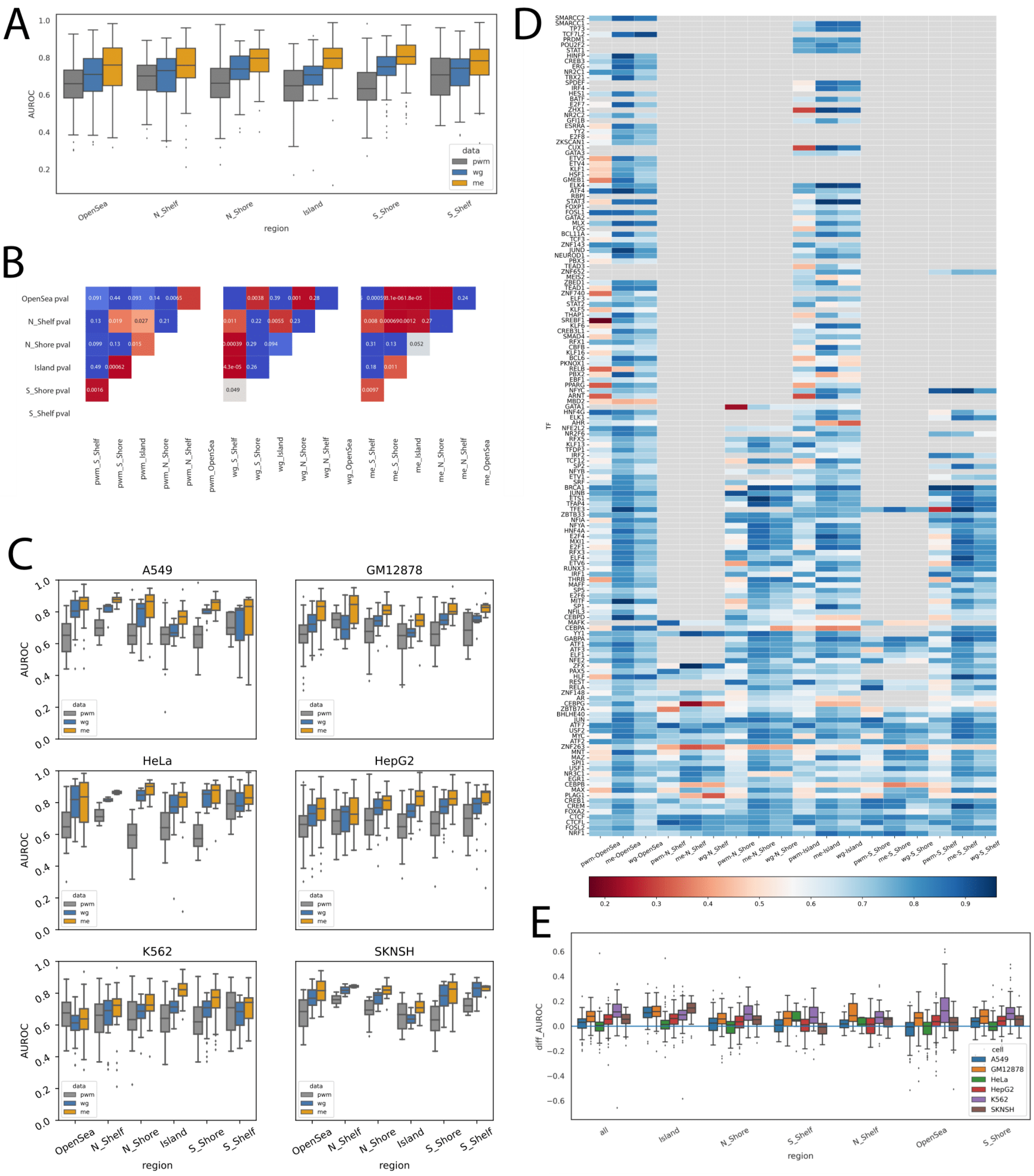

Figure S6. Detailed analysis based on genomic region. (A) Same plot as shown in **Figure 2B**. **(B)** Statistical analyses comparing the distribution of AUROC scores for each pair of regions when scoring motif locations using PWM, WGBS, or methylation array. Shades of red indicate significant differences based on a T-test and associated p-value. **(C)** Cell line specific versions of panel (A). **(D)** Heatmap of specific TF performance averaged across cell lines per genomic region annotation. Blue indicates an AUC>0.5 while red indicates AUC<0.5. **(E)** Differential AUROC between methylation array and WGBS indicating that scoring based on methylation array generally performs better than scoring based on the WGBS data.

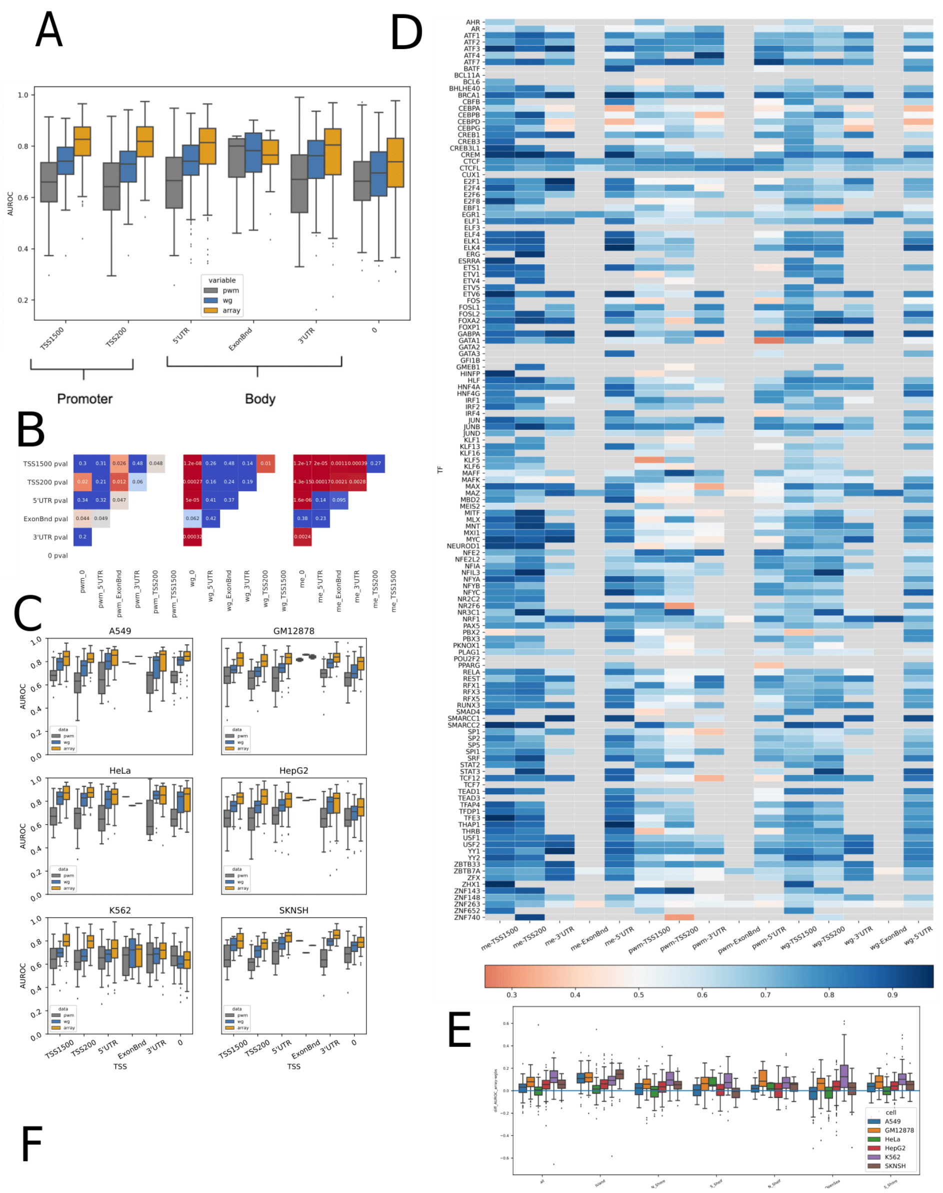

F

methyl	array							wg						
**region**	**body**						**promoter**	**body**						**promoter**
**cell**	**A549**	**HepG2**			**K562**		**K562**	**GM12878**	**HepG2**			**K562**		**K562**
**TF**	**CEBPB**	**CEBPA**	**CEBPD**	**CEBPG**	**CEBPD**	**CEBPG**	**PBX2**	**CEBPB**	**CEBPA**	**CEBPD**	**CEBPG**	**CEBPD**	**CEBPG**	**PBX2**
**pwm**	0.464354	0.488964	0.491505	0.572996	0.576504	0.597121	0.500639	0.823875	0.488964	0.491505	0.572996	0.576504	0.597121	0.500639
**auc**	0.447948	0.400495	0.444960	0.476629	0.380056	0.357290	0.439336	0.496086	0.404890	0.372503	0.415761	0.403233	0.435998	0.372925

Figure S7. Detailed analysis based on TSS annotations. (A) Same plot as shown in **Figure 2C**. **(B)** Statistical analyses comparing the distribution of AUROC scores for each pair of TSS annotations when scoring motif locations using PWM, WGBS or methylation array. Shades of red indicate significant differences based on a T-test and associated p-value. **(C)** Cell line specific versions of panel (A). **(D)** Heatmap of specific TF performance averaged across cell lines per TSS annotation. Blue indicates an AUC>0.5 while red indicates AUC<0.5. **(E)** Differential AUROC between methylation array and WGBS indicating that scoring based on methylation array generally performs better than scoring based on WGBS data. **(F)** Transcription factors whose context- and cell line specific binding is better predicted using the PWM compared to methylation data and whose methylation-based predictive performance has an AUROC <0.5. Many of these are CEBP family members.

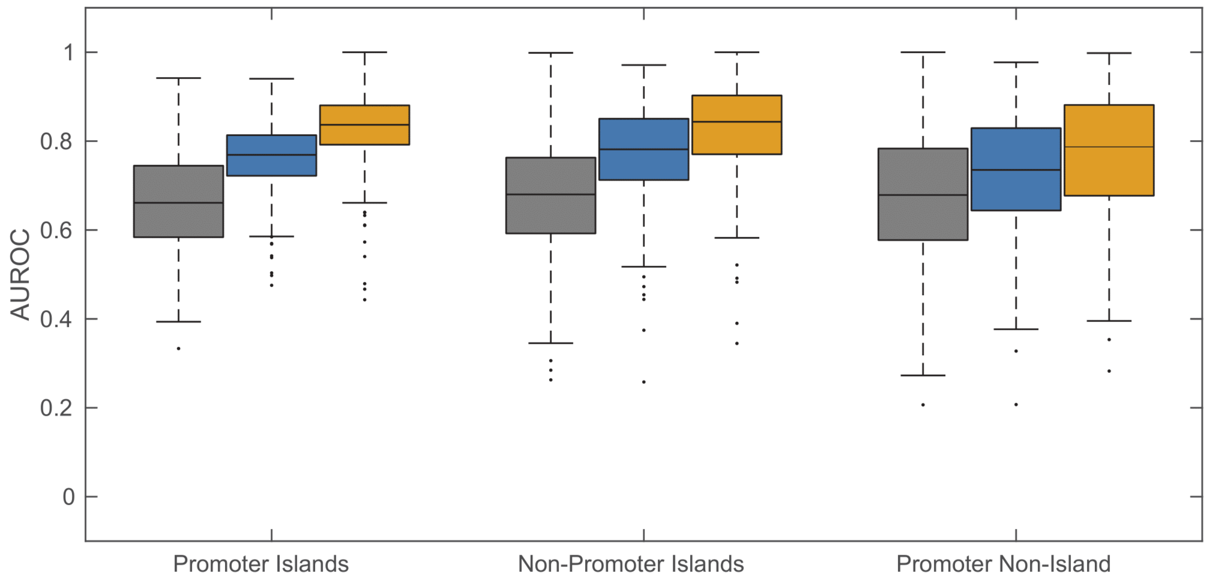

Figure S8: Distribution of AUROC scores when scoring motif locations using CpGs annotated to (1) both CpG Islands (Islands/Shores/Shelves) and gene promoters (TSS200 or TSS1500), (2) CpG Islands and not gene promoters, and (3) gene promoters but not CpG Islands.

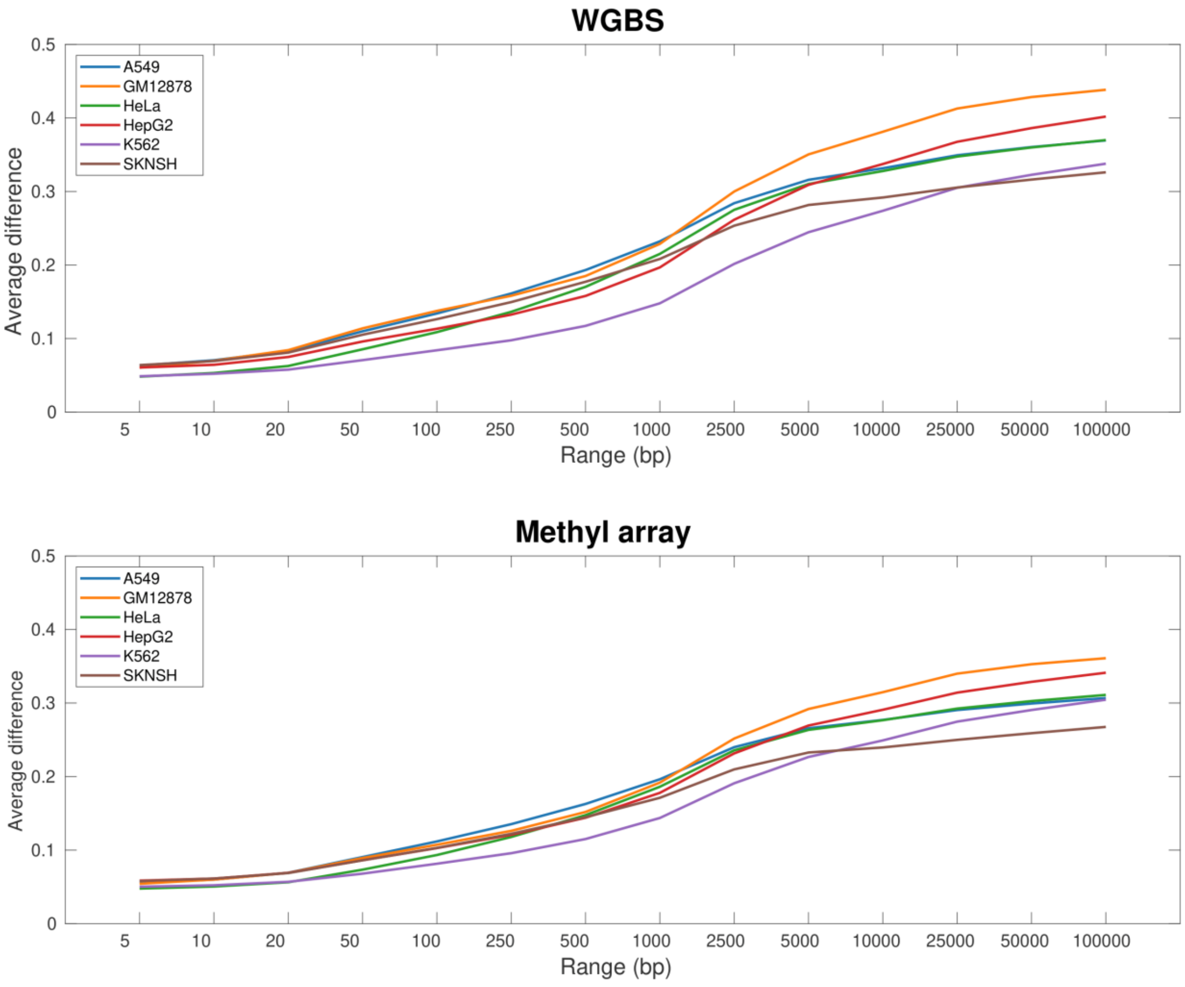

Figure S9: Average difference in the β values for all pairs of CpGs within a given range. For consistency with the main text, only CpGs that were assayed by both methylation array and in WGBS with a read depth of at least 10 were used.
